# Study on PD-1 inhibitor combined with recombinant human endostatin and chemotherapy followed by IMRT in the treatment of advanced NSCLC

**DOI:** 10.1097/MD.0000000000041306

**Published:** 2025-02-07

**Authors:** FaQiang Ma, ZhengJun Qi, GuangHui Liao, LiLi Zhao, XiaLu Su, ChangFen Dong, FangYang Lu, Yi Sun

**Affiliations:** a Department of Oncology, The Second Affiliated Hospital of Guizhou Medical University, Kaili, Guizhou, China; b Department of Oncology, Guizhou Provincial People’s Hospital, Guiyang, Guizhou, China.

**Keywords:** advanced non-small cell lung cancer, chemotherapy, PD-1 inhibitor, recombinant human endostatin

## Abstract

Although chemotherapy, targeted therapy, and antiangiogenic drugs have become the cornerstones of treatment for advanced non-small-cell carcinoma (NSCLC) in clinical practice, the emergence of immune checkpoint inhibitors (such as PD-1 inhibitors) in recent years has also provided new options for the treatment of NSCLC. To explore whether PD-1 inhibitors combined with recombinant human endostatin and chemotherapy followed by IMRT have a certain curative effect in the treatment of advanced NSCLC. We retrospectively analyzed 47 patients with stage IIIB, IIIC, and IV NSCLC admitted to our hospital from August 2022 to June 2023. According to the treatment method, the patients were divided into an observation group (24 cases) and a control group (23 cases). The control group only received recombinant human endostatin 105 g and chemotherapy followed by IMRT; the observation group received domestic PD-1 inhibitors, 200 mg, and chemotherapy followed by IMRT on the basis of the treatment plan of the control group. After treatment, the objective response rate (ORR), disease control rate, overall survival, progression-free survival (PFS), duration of response, and incidence of adverse reactions were compared. After treatment, the ORR and disease control rate of the observation group were higher than those of the control group; compared with the control group, the PFS, overall survival, and duration of response period of the observation group were longer (*P* < .05); the incidence of adverse reactions in the observation group was significantly lower than that in the control group (*P* < .05). After chemotherapy, the CD3^+^ and CD4^+^ index of the observation group was significantly increased, the CD3^+^ CD8^+^ was slightly lower than that of the control group, without statistical significance, and the ratio of CD4/CD8 was higher than that of the control group, and the interleukin-2 index was significantly better than that of the routine group, *P* < .05. PD-1 inhibitor combined with recombinant human endostatin and chemotherapy followed by IMRT in the treatment of advanced NSCLC can significantly improve the ORR and prolong the PFS of patients, and the adverse reactions are controllable. The results of our study may provide help for the treatment strategies of patients with refractory advanced NSCLC, and are worthy of promotion and use.

## 1. Introduction

Clinically, about 70% of NSCLC patients are diagnosed as having locally advanced or metastatic disease that is not suitable for surgical resection; in patients with advanced non-small-cell carcinoma (NSCLC), drug therapy plays an important role. However, the curative effect of chemotherapy has reached a plateau, and the curative effect has not improved significantly in the past 10 to 15 years; only 30% of patients can benefit from targeted therapy. Recently, tumor immune checkpoint therapy has tardily become a research and development hotspot and has continuously achieved great breakthroughs. More and more ICIs targeting PD-1/PD-L1 have been applied to advanced NSCLC, which has led to the rapid development of the treatment of advanced NSCLC with negative driver genes.^[1,2]^ In the KEYNOTE-001 study, pembrolizumab was the first-line monotherapy for EGFR gene mutation-negative advanced NSCLC, and the 5-year survival rate was 23.2%, while the KEYNOTE-189 postgraduate study showed that the median OS can reach 22 months with chemotherapy combined with immunotherapy, and the Chinese Society of Clinical Oncology included chemotherapy combined with immunotherapy in advanced NSCLC with negative driver genes as a class I recommendation (Chinese Society of Clinical Oncology (CSCO) Guidelines for the Diagnosis and Treatment of Non-Small Cell Lung Cancer 2021, n.d.). Compared with chemotherapy, the combination therapy group was beneficial in the median progression-free survival (PFS) and median overall survival (OS) (11.3 vs 8.3 months, *P* = .0001; 27.9 vs 20.5 months, *P* = .0117); in terms of safety, reactive skin capillary hyperplasia was the most common, with an incidence rate of 78%, most of which were grade 1 to 2. In the RATIONALE307 study,^[3,4]^ patients with advanced squamous NSCLC were randomly assigned to tislelizumab combined with chemotherapy or chemotherapy alone; the PFS of the combined treatment group was significantly improved. Thanks to the above studies, PD-1 inhibitors combined with platinum-based double-drug chemotherapy have become the recommended first-line treatment for advanced NSCLC in the Chinese Society of Clinical Oncology guidelines.

Tumor angiogenesis involves many aspects, including various cellular pathways and many factors that promote and inhibit angiogenesis, and these factors together determine whether blood vessels form or not. In the TME, vascular endothelial cell growth factor (VEGF) is an important factor in tumor-associated immunosuppression. Angiogenesis and immune evasion are interdependent processes considered to be one of the hallmarks of cancer.^[5]^ Due to the rapid growth and active metabolism of tumor cells, a large amount of oxygen is required, which can easily lead to hypoxia and acidosis in the tumor bed. Subsequently, hypoxia can stimulate tumor cells to produce a large amount of angiogenic factors such as VEGF.^[6]^ The local balance of angiogenesis factors and angiogenesis inhibitors is disrupted, and multiple angiogenic pathways are activated. Neonatal tumor blood vessels are abnormal in shape, and there are large gaps between adjacent endothelial cells, which are prone to vascular leakage. Abnormal tumor angiogenesis reduces the number of adhesion molecules between endothelial cells and prevents immune cell infiltration in the TME, thereby creating an immunosuppressive microenvironment. VEGF-mediated immunosuppression provides a theoretical basis for the combined application of immunotherapy and antiangiogenic therapy in cancer patients. Antiangiogenic drugs restore tumor blood vessels to normal, improve tumor hypoxia, reduce the accumulation of immunosuppressive factors such as VEGF and regulatory T cells, reduce the accumulation of immunosuppressive substances such as adenosine and lactic acid in TME, and reverse the body’s immunosuppressive state.^[7]^ At the same time, the antitumor immune response activated by immunotherapy can normalize blood vessels, and then ICIs combined with antiangiogenesis build a positive feedback pathway.^[8,9]^ The combination of PD-1 inhibitors and antiangiogenic drugs provides a new treatment option for advanced NSCLC. However, there are too many types of ICIs and antiangiogenic drugs. How to choose the appropriate drugs and the order of administration needs to be further explored.

This combination therapy has become a new option for the treatment of advanced NSCLC and has become a research hotspot of lung cancer treatment, and there are currently many clinical trials evaluating this combination therapy. As drugs tested in clinical trials are widely used clinically, differences in efficacy are often observed, and the same treatment modality may have different effects on different crowds. With the approval of camrelizumab, sintilimab, and tislelizumab for lung cancer indications, we need to obtain real data from the real world to address the immunotherapy needs of patients under different conditions. Moreover, there is a lack of clinical data on the combination of PD-1 inhibitors and Endostar in NSCLC patients. At present, there is no study comparing the efficacy of different types of ICIs combined with Endostar, and the occurrence of adverse reactions and drug tolerance in reality needs to be further verified. This study took patients with advanced NSCLC as the research object, analyzed the prognostic factors of PD-1 inhibitors combined with recombinant human endostatin and chemotherapy followed by INRT in the treatment of NSCLC, and explored its effectiveness.

## 2. Methods and materials

### 2.1. Basic information

**Figure 1.**
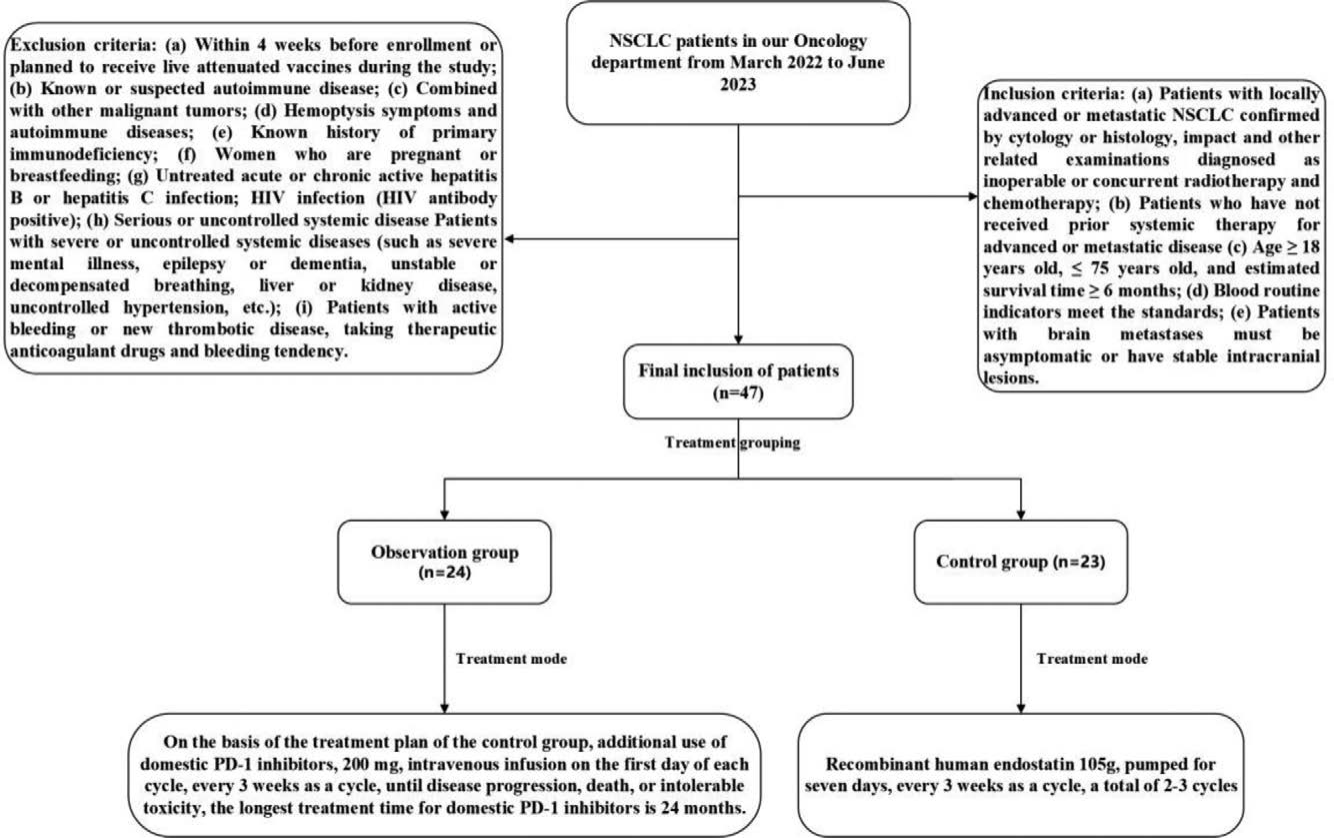
Patient inclusion flow chart.

This study was approved by the Ethics Committee of The Second Affiliated Hospital of Guizhou Medical University. From March 2022 to June 2023, a total of 47 patients with stage IIIB, IIIC, and IV NSCLC were selected from the Department of Oncology of our hospital. According to the treatment mode, the patients were classified as an observation group of 24 cases and a control group of 23 cases. The specific process is shown in Figure 1.

### 2.2. Inclusion and exclusion criteria

Inclusion criteria: patients with locally advanced or metastatic NSCLC confirmed by cytology or histology, impact, and other related examinations diagnosed as inoperable or concurrent radiotherapy and chemotherapy; patients who have not received prior systemic therapy for advanced or metastatic disease; age ≥ 18 years old, ≤ 75 years old, and estimated survival time ≥ 6 months; blood routine indicators meet the standards; and patients with brain metastases must be asymptomatic or have stable intracranial lesions.

Exclusion criteria: within 4 weeks before enrollment or planned to receive live attenuated vaccines during the study; known or suspected autoimmune disease; combined with other malignant tumors; hemoptysis symptoms and autoimmune diseases; known history of primary immunodeficiency; women who are pregnant or breastfeeding; untreated acute or chronic active hepatitis B or hepatitis C infection; HIV infection (HIV antibody positive); patients with severe or uncontrolled systemic diseases (such as severe mental illness, epilepsy or dementia, unstable or decompensated breathing, liver or kidney disease, and uncontrolled hypertension); and patients with active bleeding or new thrombotic disease, taking therapeutic anticoagulant drugs and bleeding tendency.

### 2.3. Methods

Control group: recombinant human endostatin 105 g, pumped for 7 days every 3 weeks as a cycle, a total of 2 to 3 cycles.

Observation group: On the basis of the treatment plan of the control group, additional use of domestic PD-1 inhibitors, 200 mg, intravenous infusion on the first day of each cycle, every 3 weeks as a cycle, until disease progression, death, or intolerable toxicity, the longest treatment time for domestic PD-1 inhibitors is 24 months.

All patients received pretreatment evaluations such as chest and abdomen computed tomography (CT), head magnetic resonance imaging, SPECT bone scan, or positron emission tomography-CT before enrollment. There are 1 to 3 distant metastases and 1 to 4 bone metastases. The patient is pathologically diagnosed as NSCLC by percutaneous lung biopsy, and the adenocarcinoma is confirmed to be negative for the driver gene by genetic testing. Before each treatment, patients in both groups underwent 3 routine examinations: blood biochemistry, electrocardiogram, and coagulation function and other examinations. In the immunotherapy group, thyroid function was also tested, and routine measures such as stomach protection and antiemesis were given. Each of the above programs takes 21 days as a cycle, and chest and abdomen CT, brain magnetic resonance imaging, and other examinations are performed every 2 treatment cycles. For stable and effective patients, continue to use this regimen until disease progression or unacceptable toxicity occurs. Considering the side effects of treatment, chemotherapy drugs can be delayed or reduced according to the patient’s condition, and the specific chemotherapy cycle can be adjusted according to the patient’s efficacy evaluation and tolerance. After 4 to 6 cycles of treatment, radiotherapy of 5400 to 6000 cGy, 200 cGy/f for chest lesions, conventional radiotherapy, 5600 to 6000 cGy, 200 cGy/f for brain metastases, and 4000 to 4400 cGy, 200 cGy/f for bone metastases.

### 2.4. Observation indicators

In this study, the 4th cycle after treatment was taken as the short-term curative effect evaluation time. According to the efficacy evaluation criteria for solid tumors, divided into complete remission (CR), stable disease (SD), partial response (PR), and progressive disease (PD), see Table 1 for details.

**Table 1:** Solid tumor response evaluation criteria.

Efficacy evaluation	Definition
CR	All lesions disappeared without new lesions, and the maintenance time was ≥ 4 wks
PR	The sum of the longest diameters of target lesions is reduced by ≥ 30% and maintained for ≥ 4 wks
SD	Between PR and PD
PD	The sum of the longest diameters of target lesions increases by ≥ 20%, or new lesions appear

CR = complete remission, PD = progressive disease, PR = partial response, SD = stable disease.

The study end points are objective response rate (ORR), OS, PFS, duration of response (DOR), and disease control rate (DCR). PFS was defined as the time from initiation of treatment to tumor progression or death from any cause; OS was defined as the time from initiation of treatment to all-cause death or last follow-up; DOR was defined as the time from randomization to disease progression or death in patients who achieved a complete or partial response; ORR is the percentage of CR + PR/CR + PR + SD + PD; DCR is the percentage of CR + PR + SD/CR + PR + SD + PD.

### 2.5. T cell subsets and interleukin-2 detection

After chemotherapy, interleukin-2 (IL-2) was detected and analyzed by FCM immunoassay; T cells were detected by the BD company lymphocyte subset detection kit (BD company reagent 38980, Multi TESTTM IMK Kit). According to the evaluation standard of peripheral blood immune cells in our hospital, the median level of CD4^+^ (T) % is 27% to 51%, and the median level of CD8^+^ (T)% is 15% to 44%.

### 2.6. Adverse reaction evaluation

According to the admission reexamination, the adverse reactions such as myelosuppression, gastrointestinal reactions, blood toxicity, liver and kidney function impairment, and thyroid hormone levels in each group were recorded, and the dosage was adjusted according to the adverse reactions of the patients.

### 2.7. Follow-up prognosis

Follow up all patients by telephone or electronic case, including reexamination and adverse reactions. Patients who were lost to follow-up, died of other reasons, or had no end-point events at the end of follow-up were included in the censored value processing, and the last follow-up time was usually used as the end-point time of PFS and OS.

### 2.8. Statistical method

The data in this research were analyzed by SPSS 26.0 software, including counting data and measurement data. The former is represented by “[n (%)]” and “χ^2^” is used for testing, and the latter is represented by mean ± standard deviation, and take *t* to carry out the test; if *P* < .05, it can be confirmed that there is a significance in the data difference.

## 3. Result

### 3.1. Basic information of 2 groups of patients

**Figure 2.**
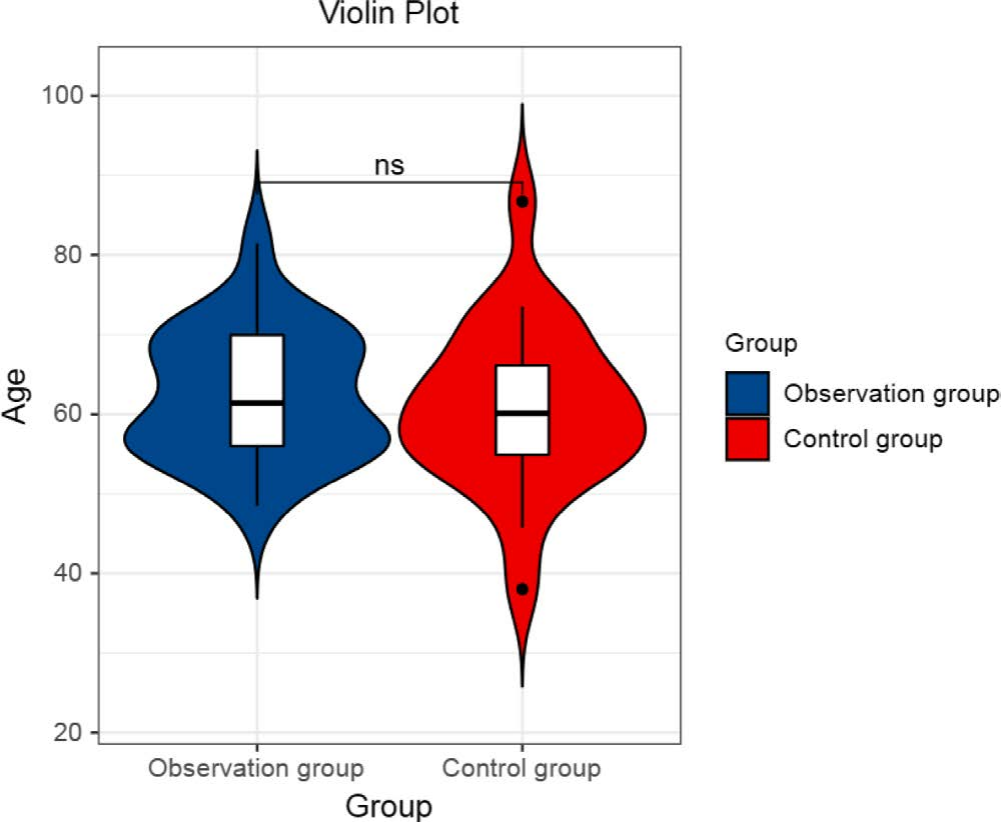
A violin plot comparing the ages. ns meant that there was no statistically significant difference between the 2 groups (*P* > .05).

First of all, in order to ensure that the general information of the 2 groups of patients is comparable, we counted the basic information of the 2 groups of patients. Among them, there were 24 people in the observation group, including 16 males and 8 females, with an average age of 62.51 ± 8.05 years old (Fig. 2). There were 23 people in the control group, including 18 males and 5 females, with an average age of 60.80 ± 10.37 years old (Fig. 2). The mean body mass index of the 2 groups was 19.6 ± 3.12 and 19.7 ± 3.21, respectively. Comparing the general information of the 2 groups of patients, the results showed that there was no statistically significant difference (*P* > .05), as shown in Table 2, indicating that the 2 groups were comparable.

**Table 2:** Gender and age comparison of patients.

Group	Male	Female	Average age	BMI
Observation group (24)	16	8	62.51 ± 8.05	19.6 ± 3.12
Control group (23)	18	5	60.80 ± 10.37	19.7 ± 3.21
*P*	.374		.530	.472

BMI = body mass index.

### 3.2. Comparison of different pathological types of patients in the 2 groups

**Figure 3.**
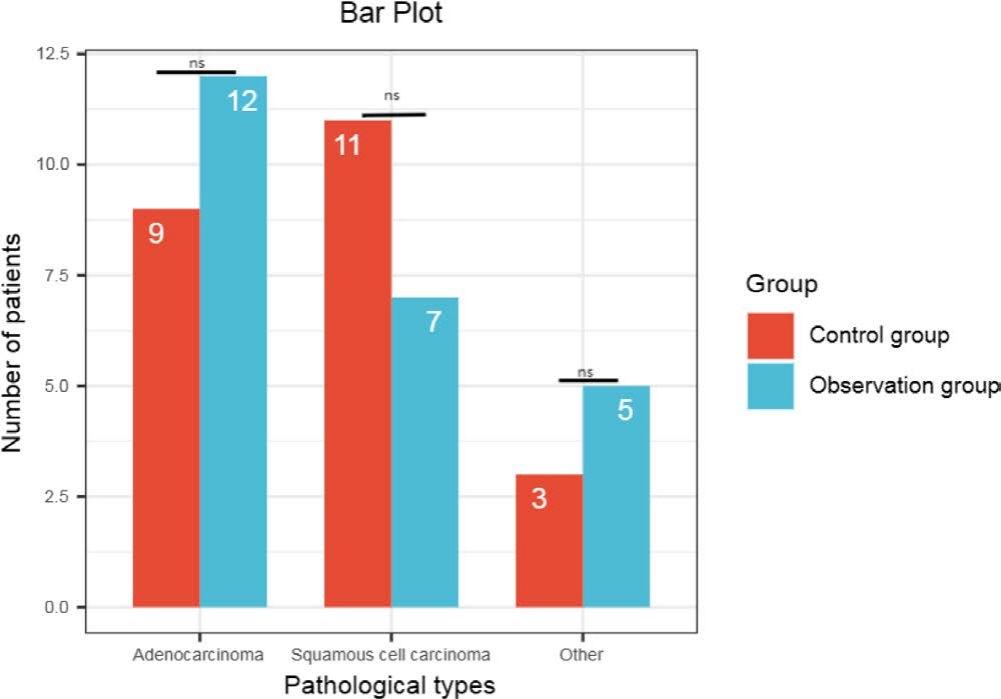
Bar plot comparing different pathology types. ns meant that there was no statistically significant difference between the 2 groups (*P* > .05).

In order to exclude the influence of different lung cancer types on the treatment effect of the 2 groups of patients, we compared the distribution of the pathological types of the 2 groups of patients. Among the 24 cases in the observation group, there were 12 cases of adenocarcinoma, 7 cases of squamous cell carcinoma, and 5 cases of others; among the 23 cases in the control group, there were 9 cases of adenocarcinoma, 11 cases of squamous cell carcinoma, and 3 cases of others. Most of the patients (adenocarcinoma and squamous cell carcinoma) and the overall distribution had no significant difference between the 2 groups (*P* > .05) (Table 3, Fig. 3). It is suggested that the main pathological type of lung cancer in this study will not be an influencing factor for the comparison of the efficacy of the 2 treatments.

**Table 3:** Comparison of different pathological types of patients in the 2 groups.

Group	Adenocarcinoma	Squamous cell carcinoma	Other
Observation group (24)	12	7	5
Control group (23)	9	11	3
*P*	.454	.188	.477

### 3.3. Comparison of curative effect between the 2 groups

**Figure 4.**
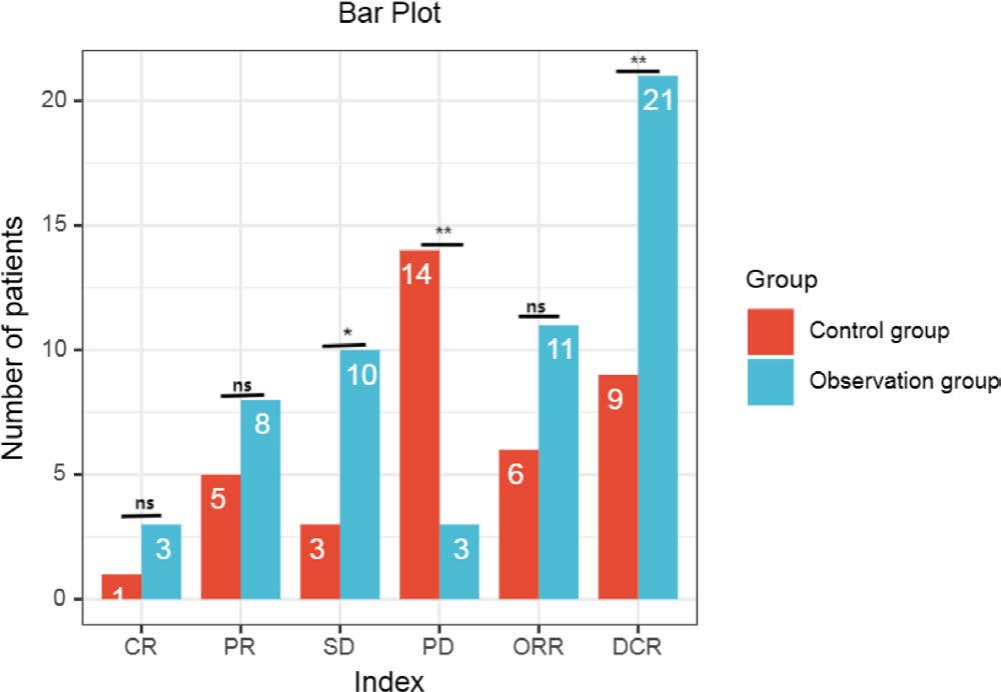
Bar plot of efficacy comparison. ns meant that there was no statistically significant difference between the 2 groups (*P* > .05). *P < .05,**P < .01. CR = complete remission, DCR = disease control rate, ORR = objective response rate, PD = progressive disease, PR = partial response, SD = stable disease.

In the 4th cycle after treatment, we compared the efficacy of the 2 groups of patients. The ORR of the observation group was slightly higher than that of the control group, and the difference between the 2 groups was not statistically significant (*P* > .05); while the DCR of the observation group was significantly higher than that of the control group, and the difference was statistically significant (*P* < .05) (Table 4, Fig. 4).

**Table 4:** Comparison of the curative effects (n [%]).

Group	CR	PR	SD	PD	ORR	DCR
Observation group (24)	3 (12.50)	8 (33.33)	10 (41.67)	3 (12.50)	11 (45.83)	21 (87.50)
Control group (23)	1 (4.35)	5 (21.74)	3 (13.04)	14 (60.87)	6 (26.09)	9 (39.13)
*t*	1.002	0.789	4.809	11.902	1.984	11.902
*P*	.317	.374	.028	.001	.159	.001

CR = complete remission, DCR = disease control rate, ORR = objective response rate, PD = progressive disease, PR = partial response, SD = stable disease.

### 3.4. Comparison of PFS, OS, and DOR between the 2 groups

**Figure 5.**
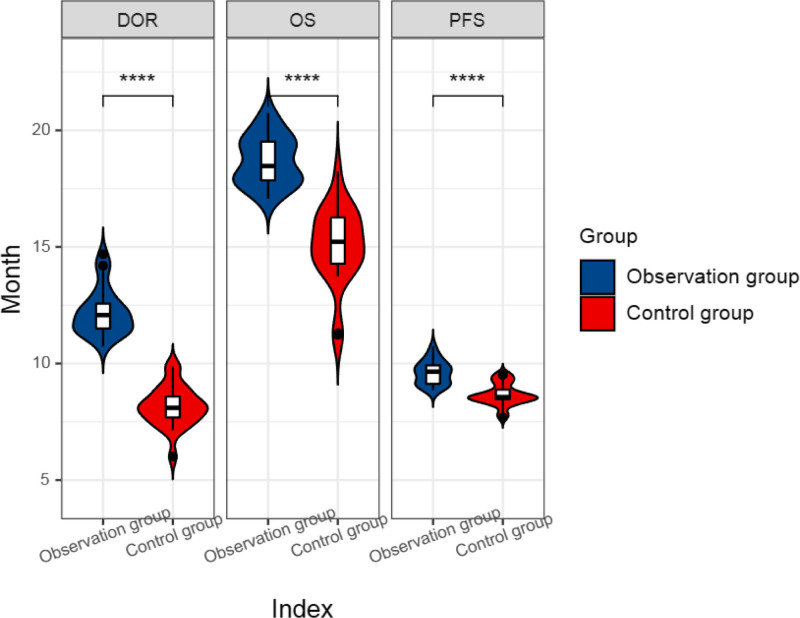
Violin plot of progression-free survival (PFS), overall survival (OS), and duration of response (DOR) comparison. ****P < .0001.

**Figure 6.**
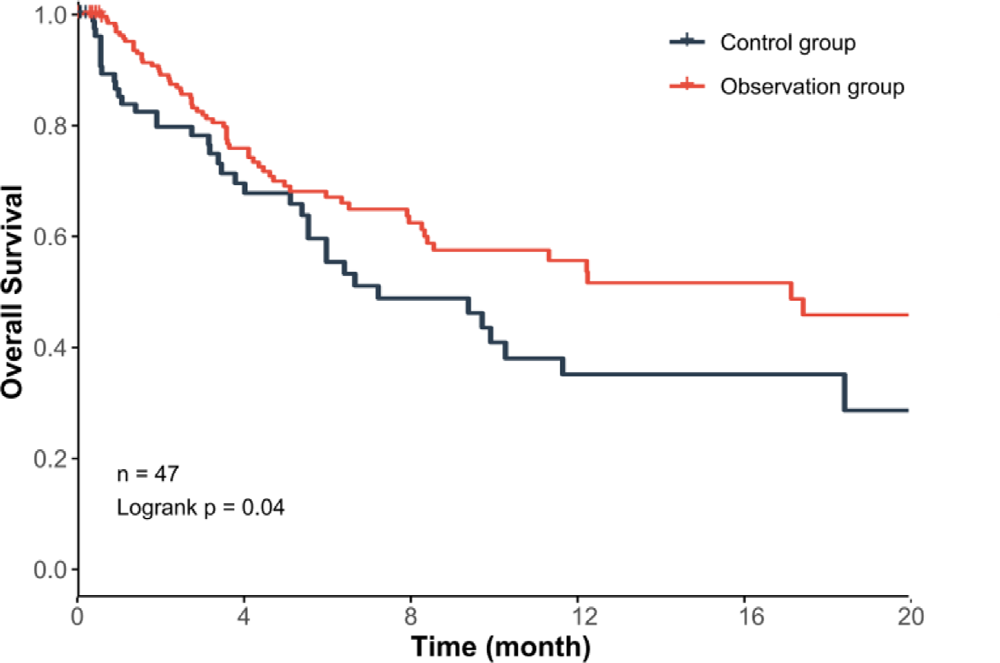
Overall survival comparison between the 2 groups.

In order to further evaluate the therapeutic effect of PD-1 inhibitors plus recombinant human vascular endostatin combined with chemotherapy followed by radiotherapy for lung lesions and oligometastatic lesions, we compared the differences in PFS, OS, and DOR between the 2 groups. The results showed that compared with the control group, the PFS, OS, and DOR periods of the observation group were longer, and the difference was statistically significant (*P* < .05) (Table 5, Fig. 5). In addition, we compared the 20-month OS survival curves of the 2 groups of patients, as shown in Figure 6, and the difference was statistically significant (*P* < .05).

**Table 5:** Comparison of PFS, OS, and DOR between the 2 groups (months).

Group	PFS	OS	DOR
Observation group (24)	9.6 ± 0.51	18.7 ± 1.05	12.2 ± 1.02
Control group (23)	8.7 ± 0.47	15.1 ± 1.63	8.2 ± 0.87
*t*	6.284	9.040	14.435
*P*	.000	.000	.000

DOR = duration of response, OS = overall survival, PFS = progression-free survival.

### 3.5. Comparison of adverse reactions

**Figure 7.**
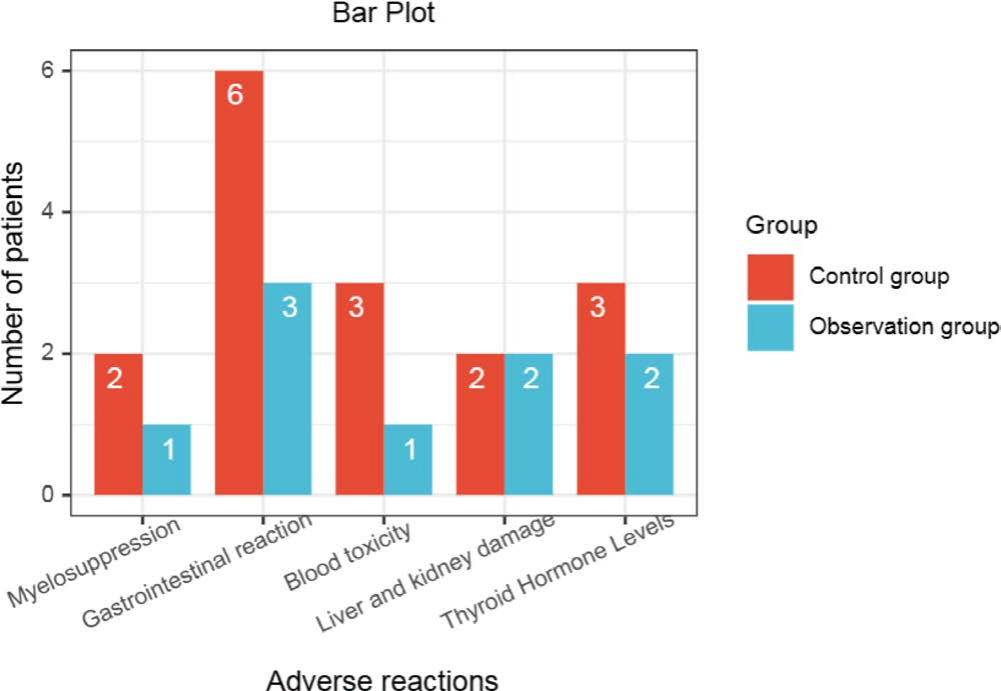
Bar plot comparing adverse reactions.

In order to observe the occurrence of common adverse drug events in the 2 groups, we statistically analyzed the incidence of several common adverse events in the 2 groups. It can be seen from the results that the incidence of adverse reactions in the observation group was significantly lower than that in the control group, and the difference was significant (*P* < .05) (Table 6, Fig. 7).

**Table 6:** Comparison of adverse reactions.

Group	Myelosuppression	Gastrointestinal reaction	Blood toxicity	Liver and kidney damage	Thyroid hormone levels	Total incidence, %
Observation group (24)	1	3	1	2	2	9 (37.50)
Control group (23)	2	6	3	2	3	16 (69.57)
*T*						4.850
*P*						.028

### 3.6. Comparison of CD4^+^ CD8^+^and IL-2 in peripheral blood

After chemotherapy, the CD4^+^, CD8^+^, and CD3^+^CD4^+^ indexes of the observation group were higher than those of the control group. However, the difference of CD3^+^CD8^+^ index between the 2 groups was not very large, and it was not statistically significant. Therefore, the immunity level of the patients in the observation group was significantly better than that in the routine group, *P* < .05. The IL-2 index of patients in the observation group was significantly better than that in the routine group, *P* < .05, and the difference was statistically significant (Table 7).

**Table 7:** Comparison of cell subsets and interleukin-2 (IL-2) in 2 groups of patients.

Group	CD4^+^	CD8^+^	CD3^+^CD4^+^	CD3^+^CD8^+^	IL-2
Observation group (24)	35.60 ± 6.35	34.592 ± 7.10	38.88 ± 8.85	29.36 ± 9.12	10.35 ± 4.42
Control group (23)	27.81 ± 6.28	30.02 ± 8.05	25.88 ± 7.58	30.16 ± 8.66	7.85 ± 3.52
*t*	14.597	9.152	11.157	1.007	10.425
*P*	.000	.000	.000	.759	.000

## 4. Discussion

NSCLC is a type of malignant lung tumor disease. Compared with small cell lung cancer, it has the characteristics of slow cell division and relatively late metastasis of cancer cells. Currently, most patients with advanced NSCLC are treated with chemotherapy.^[10]^ In recent years, studies have shown that antiangiogenic drug therapy in patients with malignant tumors can normalize tumor blood vessels, which is conducive to the effective transport of oxygen and drugs, thereby improving the sensitivity of radiotherapy and chemotherapy.^[11,12]^ At present, immunotherapy combined with chemotherapy has made great progress in NSCLC, but about 20% to 30% of the population benefited, and better treatment methods still need to be explored because the mechanisms of targeting and immunotherapy are different. Currently, there are multiple regimens of target-immunity combined with chemotherapy in research. In fact, this has a lot to do with the immunosuppressive state of the tumor microenvironment; immunotherapy and chemotherapy can change the microenvironment of tumor cells. For example, abnormal blood vessels help tumors escape the attack of the immune system, Endostar normalizes abnormal tumor blood vessels, increases the infiltration of immune effector cells, and increases CD4^+^ or IL-2, which proves immunity. The synergy of antivascular and chemotherapy can increase the curative effect, which is the reason for the high ORR in the observation group, but the adverse reactions did not increase significantly. It is expected that the long-term survival can be improved by increasing the short-term curative effect^[13]^; on the other hand, ICIs promote the normalization of blood vessels by restoring the body’s normal immune response.^[14–16]^ ICIs combined with antiangiogenic therapy can overcome ICIs resistance and improve the prognosis of patients, which is a new treatment model. In many clinical studies, ICIs combined with antiangiogenic therapy showed good antitumor ability and manageable safety in patients with advanced NSCLC.^[17,18]^ In this study, PD-1 inhibitors combined with recombinant human endostatin and chemotherapy IMRT treatment of advanced NSCLC significantly improved the PFS and ORR of patients. There was no difference between the total adverse reactions and the control group, and only the incidence of individual immune-related adverse reactions was higher than that of the control group. Although PD-1 inhibitors combined with antivascular therapy and chemotherapy can bring good clinical curative effects to patients, some patients cannot tolerate them considering the combined toxic and side effects of drug addiction, and chemotherapy is the most toxic treatment among them; follow-up studies can be developed toward the chemotherapy model. Due to the intricate relationship between the tumor microenvironment, angiogenesis, and immune response, how to select the types of immunotherapy and antiangiogenic drugs and clarify the dosage and sequence of drugs also needs to be explored on a larger scale.

This study has several limitations. In our cohort study, data including PD-L1 TPS and tumor mutation burden were not collected; considering the high cost of testing and the fact that the testing items are not included in medical insurance, most patients are unwilling to do the testing, which makes it impossible for us to know the correlation between the level of PD-L1 stratification and the efficacy of combined immune therapy. In fact, these biomarkers are currently under intense research; studies have shown that immunotherapy has clinical benefits for patients with high expression of PD-L1 and high tumor mutation burden, which are potential factors for identifying patients suitable for immunotherapy^[19–21]^; however, we still know too little about the predictive factors for the efficacy of immunotherapy combined with antiangiogenesis. With the wide application of molecular detection technologies such as genetic testing, cancer treatment has gradually evolved into individualized and precise treatment. Identification of specific gene targets in combination with immunotherapy and antiangiogenic therapy is an important goal for clinicians.^[22–25]^ Second, the follow-up time is not enough, and it is necessary to extend the follow-up time to obtain more mature OS data. In addition, the driver gene status of some patients in our study is unknown, which may interfere with the real results. Whether the use of immune combined with antivascular drugs after resistance to targeted drugs in patients with positive driver genes is better than other treatment options needs further research and exploration. Finally, as the sample size of a retrospective study is not large enough, it is inevitable that there will be recall bias and measurement bias. Therefore, more studies, especially large-sample randomized controlled clinical studies, are needed to verify the conclusions.

All in all, in this retrospective study, we observed that PD-1 inhibitors combined with recombinant human endostatin and chemotherapy IMRT can improve the short-term efficacy of patients, prolong PFS and OS, and the adverse reactions are controllable. Our research results may be refractory, and the treatment strategies of patients with advanced NSCLC can help and provide valuable clues for further large-sample, multicenter, and prospective studies in the future, so as to bring further benefits to patient survival.

## Author contributions

**Conceptualization:** Faqiang Ma, Zhengjun Qi, Yi Sun.

**Data curation:** Faqiang Ma, Zhengjun Qi, Guanghui Liao, Lili Zhao, Changfen Dong, Fangyang Lu, Yi Sun.

**Formal analysis:** Faqiang Ma, Guanghui Liao, Xialu Su, Changfen Dong, Yi Sun.

**Investigation:** Faqiang Ma, Zhengjun Qi, Xialu Su, Fangyang Lu.

**Methodology:** Faqiang Ma, Zhengjun Qi, Guanghui Liao, Lili Zhao, Xialu Su, Changfen Dong, Fangyang Lu.

**Writing – original draft:** Faqiang Ma, Yi Sun.

**Writing – review & editing:** Faqiang Ma, Yi Sun.

**Supervision:** Zhengjun Qi.

**Validation:** Lili Zhao, Xialu Su.

**Funding acquisition:** Yi Sun.
